# GRASPs in Golgi Structure and Function

**DOI:** 10.3389/fcell.2015.00084

**Published:** 2016-01-06

**Authors:** Xiaoyan Zhang, Yanzhuang Wang

**Affiliations:** ^1^Department of Molecular, Cellular and Developmental Biology, University of MichiganAnn Arbor, MI, USA; ^2^Department of Neurology, University of Michigan School of MedicineAnn Arbor, MI, USA

**Keywords:** GRASP55, GRASP65, Golgi stack, Golgi ribbon, protein glycosylation

## Abstract

The Golgi apparatus is a central intracellular membrane organelle for trafficking and modification of proteins and lipids. Its basic structure is a stack of tightly aligned flat cisternae. In mammalian cells, dozens of stacks are concentrated in the pericentriolar region and laterally connected to form a ribbon. Despite extensive research in the last decades, how this unique structure is formed and why its formation is important for proper Golgi functioning remain largely unknown. The Golgi ReAssembly Stacking Proteins, GRASP65, and GRASP55, are so far the only proteins shown to function in Golgi stacking. They are peripheral membrane proteins on the cytoplasmic face of the Golgi cisternae that form *trans*-oligomers through their N-terminal GRASP domain, and thereby function as the “glue” to stick adjacent cisternae together into a stack and to link Golgi stacks into a ribbon. Depletion of GRASPs in cells disrupts the Golgi structure and results in accelerated protein trafficking and defective glycosylation. In this minireview we summarize our current knowledge on how GRASPs function in Golgi structure formation and discuss why Golgi structure formation is important for its function.

## Introduction

The Golgi apparatus is a membrane-bound organelle found in all eukaryotic cells, including those of animals, plants, and fungi, and functions as a central hub in the exocytic secretory pathway (Klute et al., 2011). The Golgi is the receiver of the entire output of the endoplasmic reticulum (ER), where proteins and lipids are processed, sorted, and packaged into vesicles and transport carriers for delivery to their final destinations inside or outside of the cell. Under electron microscope (EM), the Golgi displays as stacks of flattened cisternae, which are often laterally linked into a ribbon-like structure in mammalian cells. By light microscopy, the Golgi is characterized by a compact reticular appearance located adjacent to the nucleus. Despite the complexity, the Golgi structure is highly dynamic, and undergoes rapidly disassembly and reassembly during mitosis and under stress and physiological conditions (Wang and Seemann, 2011). At the onset of mitosis, the Golgi disassembles into vesicles and tubular structures that are partitioned into the daughter cells, where they are reassembled into a new Golgi at the end of mitosis (Shorter and Warren, 2002).

**Figure 1:**
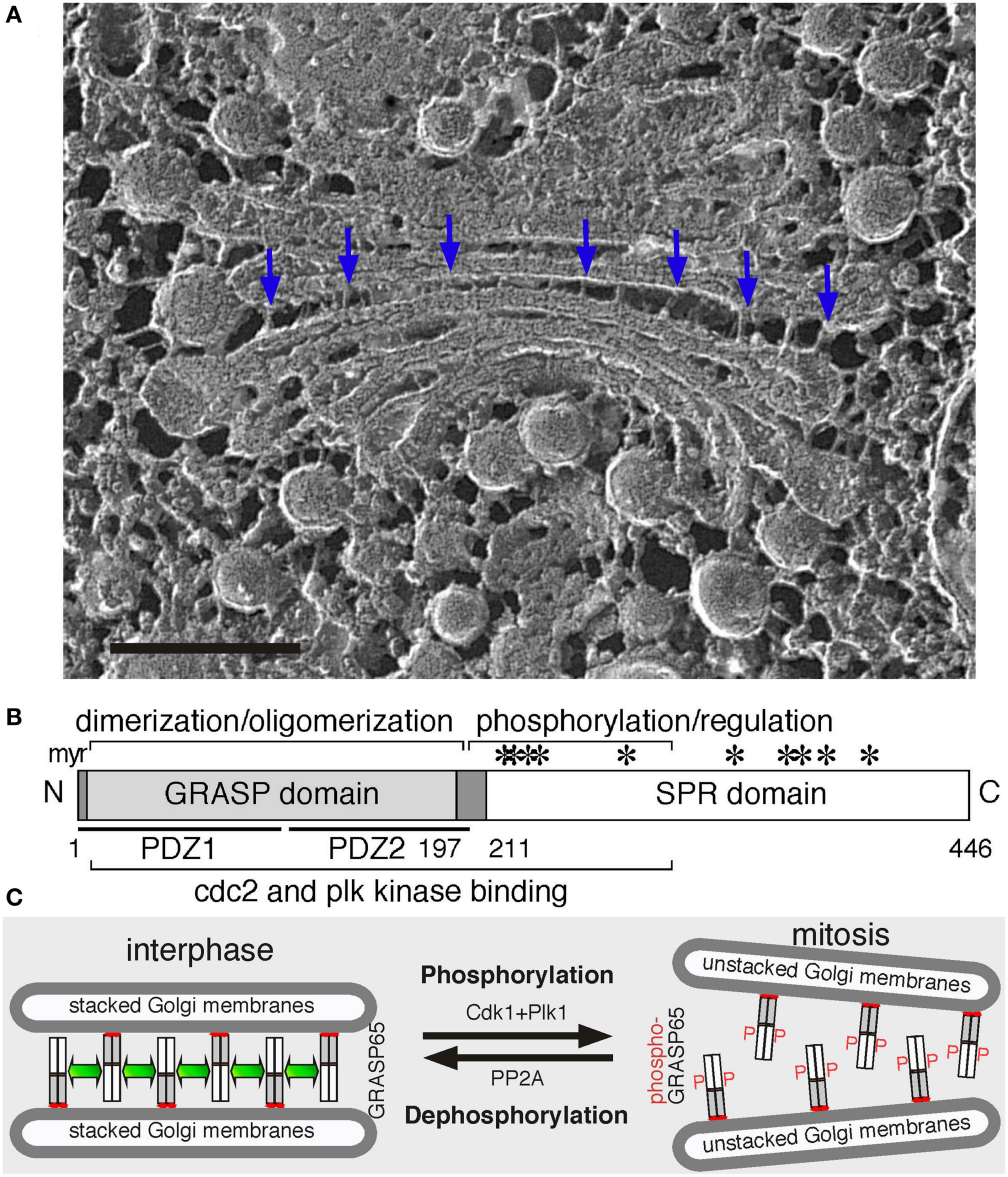
**Golgi structure and the role of GRASP65 in Golgi stack formation. (A)** Electron micrograph of a Golgi apparatus from the green alga *Chlamydomonas reinhardtii*. Cells were snap-frozen without chemical fixation, fractured and deep-etched (Heuser, 2011). Arrows point to bridges between the cisternae. Scale bar, 100 nm. **(B)** GRASP65 schematic domain structure. Indicated are the myristic acid (myr) for membrane association, the GRASP domain (with two PDZ domains underlined) for dimerization and oligomerization, and the Serine/Proline-Rich (SPR) domain with phosphorylation sites (^*^). GRASP55 has a similar domain structure. **(C)** GRASP65 oligomerization and Golgi stack formation. During interphase, GRASP65 dimers from adjacent cisternae oligomerize to form a “glue” to hold the membranes into a stack. In mitosis, phosphorylation of GRASP65 by Cdk1 and Plk1 disassembles the oligomers and unstacks the cisternae. Post-mitotic dephosphorylation of GRASP65 by PP2A leads to re-oligomerization.

The unique stacked morphology and dynamics of the Golgi have prompted numerous studies targeting the mechanisms of Golgi structure formation and function. Morphological and biochemical research observed inter-cisternal proteinaceous connections that cross-link adjacent cisternae (Figure 1A) (Turner and Whaley, 1965; Franke et al., 1972; Heuser, 2011). Mild proteolysis removing these connections resulted in unstacking (Cluett and Brown, 1992). Later on, a detergent-insoluble protein complex was isolated, suggesting the presence of a “Golgi matrix” to which Golgi enzymes could attach (Slusarewicz et al., 1994). Numerous Golgi matrix proteins have been identified and characterized since then, including GRASPs (GRASP55 and GRASP65) and golgins, which work together to maintain Golgi structure and function (Xiang and Wang, 2011).

GRASP65 was first discovered as a Golgi stacking protein that is accessible to the alkylating reagent *N-ethylmaleimide* (NEM) only when the Golgi stack is disassembled (Barr et al., 1997). It is a peripheral protein on the cytoplasmic surface of the Golgi, directly targeted to the Golgi after synthesis in the cytosol (Yoshimura et al., 2001) via a myristic acid attached to the N-terminal glycine residue. In a cell-free system that mimics Golgi disassembly and reassembly during the cell cycle, inhibition of GRASP65 using recombinant proteins or antibodies blocked the formation of Golgi stacks but not the generation of single cisternae (Barr et al., 1997). When cells were treated with Brefeldin A (BFA), a fungal metabolite that redistributes Golgi enzymes into the ER (Orci et al., 1991; Klausner et al., 1992), GRASP65 and GM130 remain in small tubulovesicular remnants distinct from the ER, which function as the receiver of Golgi membranes upon BFA washout (Seemann et al., 2000). The GRASP65-GM130 complex also functions as a Rab1 effector to define the *cis*-Golgi compartment that receives COPII vesicles from the ER (Moyer et al., 2001).

GRASP55 was identified as a homolog of GRASP65 by database searching (Shorter et al., 1999). Similar to GRASP65, GRASP55 interacts with Golgin-45 and Rab2, and is essential for protein transport and Golgi structure formation (Short et al., 2001; Barr, 2005). The proposed role for both GRASPs in Golgi stacking was potentiated by their subcellular localization. Cryo-EM revealed that GRASP65 is present in *cis*-Golgi, while GRASP55 is more concentrated in the *medial/trans*-cisternae (Shorter et al., 1999). Thus, although GRASP55 and GRASP65 may have some redundancy in their functions, they are both required for the formation of the polarized stacked structure (Xiang and Wang, 2010).

GRASPs are evolutionally conserved. Both contain an N-terminal GRASP domain, which is highly conserved between the two and between species, and a C-terminal Serine/Proline-Rich (SPR) domain, which is more divergent (Figure 1B). GRASP orthologues and homologs have been identified in different species, including flies (Kondylis et al., 2005), yeast (Behnia et al., 2007), and parasites (Ho et al., 2006; Struck et al., 2008; Yelinek et al., 2009), but not in plants (Vinke et al., 2011). These homologs have a higher sequence homology to mammalian GRASP55 than GRASP65, suggesting that GRASP55 may be the common ancestor during evolution. Most of these homologs are associated with the Golgi; however, some are also detected on other membranes. For instance, the sole GRASP homolog in *Drosophila melanogaster*, dGRASP, localizes to the Golgi and the transitional ER (tER); depletion of dGRASP in S2 cells by RNA interference (RNAi) partially affected the Golgi structure but had no effect on tER organization (Kondylis et al., 2005). In the budding yeast *Saccharomyces cerevisiae*, which has most of the Golgi cisternae as isolated discs, the single GRASP homolog Grh1 localizes to the tER-Golgi interface but is not required for tER-Golgi association (Behnia et al., 2007; Levi et al., 2010). Thus, GRASPs function in Golgi stacking may be a gained function during evolution.

So far GRASP65 and GRASP55 are the only known proteins with the properties required for Golgi stacking. A number of labs have tested these proteins; some support their roles in Golgi stacking, while others provided alternative functions including Golgi ribbon linking, transport of specific cargo across the Golgi stack, unconventional secretion, cell cycle regulation, apoptosis, and cell migration, which have been summarized in a number of reviews (Wang, 2008; Ramirez and Lowe, 2009; Wei and Seemann, 2010; Vinke et al., 2011; Wang and Seemann, 2011; Xiang and Wang, 2011; Ji et al., 2013; Tang and Wang, 2013). Most recently, new findings have been made on GRASPs, including available crystal structures, identification of novel GRASP interacting proteins, and new insights between Golgi structure formation and function, which have triggered us to update our understanding of GRASPs in Golgi structure formation and function. Other functions of GRASPs not related to the Golgi are not discussed here due to space limitations.

## GRASPs and golgi structure formation

GRASPs have the biochemical and biophysical properties to function as Golgi stacking proteins. First, GRASPs are peripheral proteins located between the cisternae where stacking occurs (Shorter et al., 1999). In addition to N-myristoylation, GRASP65, and GRASP55 also interacts with GM130 (Barr et al., 1998) and Golgin-45 (Short et al., 2001), respectively. This dual anchoring of GRASPs onto membrane restricts the orientation of the protein to favor *trans* pairing over *cis* (Bachert and Linstedt, 2010), thus ensuring membrane tethering by forming *trans*-oligomers (Wang et al., 2003).

Second, GRASPs oligomerization is regulated by phosphorylation, which provides an explanation for Golgi disassembly and reassembly during cell division (Tang and Wang, 2013). In cells, inhibition of mitotic kinases blocked mitotic Golgi fragmentation (Misteli and Warren, 1995); while microinjection of mitotic kinases such as Cdk1 and polo-like kinase (Plk) led to Golgi disassembly (Wang et al., 2003). *In vitro*, treatment of purified Golgi stacks with mitotic kinases resulted in cisternal unstacking (Wang et al., 2003; Tang et al., 2008). These results demonstrate that Golgi structure formation is regulated by phosphorylation during the cell cycle. GRASP65 is a major target of mitotic kinases on the Golgi (Wang et al., 2003); the SPR domain contains multiple phosphorylation sites that are phosphorylated by Cdk1 and Plk in mitosis (Tang et al., 2012), which inhibits GRASP oligomerization and results in Golgi disassembly (Wang et al., 2005). At the end of mitosis, GRASP65 dephosphorylation by PP2A (Tang et al., 2008) allows the reformation of GRASP *trans*-oligomers and restacking of newly formed cisternae (Tang et al., 2010). GRASP55 is regulated in a similar way (Xiang and Wang, 2010), though phosphorylated by the MAP kinase ERKs instead (Jesch et al., 2001; Feinstein and Linstedt, 2007; Duran et al., 2008).

Third, the size of GRASP proteins fits the tight gap between the cisternae. Recently reported crystal structures confirmed that the GRASP domain is globular, with 6.5 nm in length, and that this domain forms oligomers (Truschel et al., 2011; Feng et al., 2013; Hu et al., 2015). There are some differences between these reports on the arrangements of the GRASP domain, possibly because of the differences in the protein length used in the studies and the addition of a GM130 peptide that may cause conformational change. None of the structural studies were able to include the SPR domain, and thus the structural basis of phosphorylation regulation of GRASP oligomerization remains unknown. Nevertheless, the size of GRASP65 *trans*-oligomers fits well with the 11 nm inter-cisternal gap (Cluett and Brown, 1992). These results suggest GRASPs as ideal candidates in Golgi stacking than the long coiled-coil golgins, which are better known for membrane tethering (Wong and Munro, 2014).

Since their discoveries, additional evidence has been provided to support GRASPs as Golgi stacking factors. Biochemical studies revealed that GRASP65 forms homodimers through the GRASP domain; dimers from adjacent membranes oligomerize in *trans* and *trans*-oligomers function as a “glue” to hold the cisternae together into stacks (Figure 1C) (Wang et al., 2003). When GRASP65 is coated onto the surface of beads, it causes the beads to aggregate, demonstrating that it can directly link surfaces together (Wang et al., 2003, 2005). Similarly, expressing GRASP65 on the outer membrane of mitochondria led to mitochondria aggregation (Sengupta et al., 2009). In cells, microinjection of GRASP65 antibodies inhibited post-mitotic Golgi reassembly (Wang et al., 2003). Depletion of either GRASP by RNAi reduced the number of cisternae per stack (Sütterlin et al., 2005), which was rescued by expressing exogenous GRASP proteins (Tang et al., 2010). Simultaneous depletion of both caused complete disassembly of the Golgi stacks (Xiang and Wang, 2010). Conversely, expression of non-phosphorylatable GRASP65 mutants enhanced Golgi stacking in interphase and inhibited Golgi fragmentation in mitosis (Tang et al., 2010). Similar results were obtained for GRASP55 (Xiang and Wang, 2010).

RNAi-mediated knockdown has been widely used to investigate the role of GRASPs in Golgi structure formation. A recent study from the Rothman lab showed that efficient stacking occurs in the absence of GRASP65/55 when either GM130 or Golgin-45 was overexpressed, and thus hypothesized that a large number of proteins, including GRASP55/65, Golgin-45, GM130, and perhaps additional proteins, contribute to the total amount of adhesive energy that glues Golgi cisternae into a stack (Lee et al., 2014). While this hypothesis indicates a high complexity in Golgi stacking, it helps explain how Golgi stacking occurs in organisms such as plant in which no GRASP proteins have been identified. Consistent with this hypothesis, knockdown of both dGRASP and GM130 in *Drosophila* S2 cells resulted in more dramatic Golgi disassembly than depletion of dGRASP alone (Kondylis et al., 2005). A GRASP65 knockout mouse has recently been reported, with only limited defects in Golgi structure and function (Veenendaal et al., 2014). A concern with this mouse is that a functional fragment of GRASP65 may still be made, and the knockout effect of GRASP65 may be compensated by the redundancy of GRASP55. Therefore, a complete knockout of both GRASPs is needed to further evaluate their functions.

In some other reports, RNAi-mediated depletion of GRASP65 or GRASP55 also resulted in Golgi ribbon unlinking, but the Golgi stacks were largely intact, suggesting that GRASPs may link Golgi stacks into a ribbon (Puthenveedu et al., 2006; Feinstein and Linstedt, 2008). While the different observations may be due to distinct experimental systems, the knockdown efficiency, and the approaches used to analyze the effects of GRASP deletion. In fact, these two observations are not mutually exclusive, and it is possible that GRASPs function in both Golgi stacking and ribbon linking by forming *trans*-oligomers.

Given that the gaps between Golgi stacks are much larger and more heterogeneous (10s–100s nm) than the distance between cisternae within each stack (Cluett and Brown, 1992), it is possible that other bridging proteins may help GRASPs in ribbon linking, of which golgins are ideal candidates because of their long coiled-coil domains known in membrane tethering. Consistent with this idea, inhibition (by RNAi-mediated depletion or microinjection of antibodies) of GM130 (Puthenveedu et al., 2006), Golgin-84 (Diao et al., 2003), Golgin-97 (Lu et al., 2004), Golgin-160 (Maag et al., 2005), and p115 (Chiu et al., 2002), all results in fragmentation of the Golgi ribbon into ministacks (Munro, 2011). An ideal bridge protein for GRASP65 is GM130 (Barr et al., 1998; Nakamura, 2010); however, the level and localization of GM130 are not significantly affected by GRASP65 depletion (Sütterlin et al., 2005; Tang et al., 2010), indicating a role for GM130 in Golgi integrity independent of GRASP65.

To explore the possibility that other proteins may help GRASP65 in ribbon linking, we have employed biochemical methods and identified the actin elongation factor Mena as a novel GRASP65 binding protein (Tang et al., 2016). Mena is recruited onto the Golgi membranes through interaction with GRASP65 and triggers local actin filament growth. Depletion of Mena or disrupting actin polymerization resulted in Golgi fragmentation. In cells, Mena and actin were required for Golgi ribbon formation after nocodazole washout; *in vitro*, Mena, and microfilaments enhanced GRASP65 oligomerization and Golgi membrane fusion. Thus, Mena interacts with GRASP65 to promote local actin polymerization and GRASP65 oligomerization, both of which facilitate Golgi ribbon linking.

## GRASPs and Golgi function

To a great extent, organelle function relies on its structure. However, why Golgi stack formation is important for its function has been remaining largely as a mystery in the field for many decades. Golgi cisternae do not normally form stacks in budding yeast (*Saccharomyces cerevisiae*), suggesting that stacking is not absolutely required for cell survival. However, Golgi stacking is a pronounced feature in all metazoans and many unicellular eukaryotes, implying that it has important functional consequences. First, stacking may impact protein trafficking. The close spatial arrangement of cisternae in stacks minimizes the distance that molecules must travel; local tethering proteins facilitate vesicle fusion with Golgi membranes (Lupashin and Sztul, 2005), therefore stacking should enhance protein trafficking. However, stacking restricts the surface for vesicle budding and fusion to the rims of the cisternae and so it may retard trafficking. Thus, this relationship is still not well understood. Second, stacking may be required for accurate glycosylation. The Golgi harbors various glycosyltransferases and glycosidases in different sub-compartments. An ordered structure is likely required to carry out precise, sequential modifications as cargo proteins pass between cisternae (Kornfeld and Kornfeld, 1985; Varki, 1998; Roth, 2002). In yeast and other fungi, N-glycosylation in the Golgi mainly involves the addition of mannoses (Wildt and Gerngross, 2005). In multi-cellular organisms, N-glycosylation of membrane and secretory proteins is more complex and critical. Accurate glycosylation is essential for their cellular functions, including cell adhesion and migration, cell-cell communication, and immunity (Ohtsubo and Marth, 2006). In polarized cells such as neurons and epithelial cells, N- and O-linked glycosylations serve as apical sorting signals (Weisz and Rodriguez-Boulan, 2009). This may explain why stacking is not required in yeast, but is essential for life in higher order organisms. Third, stacking may ensure that sorting occurs only when cargo molecules reach the *trans*-Golgi network (TGN) but not in earlier sub-compartments.

**Figure 2:**
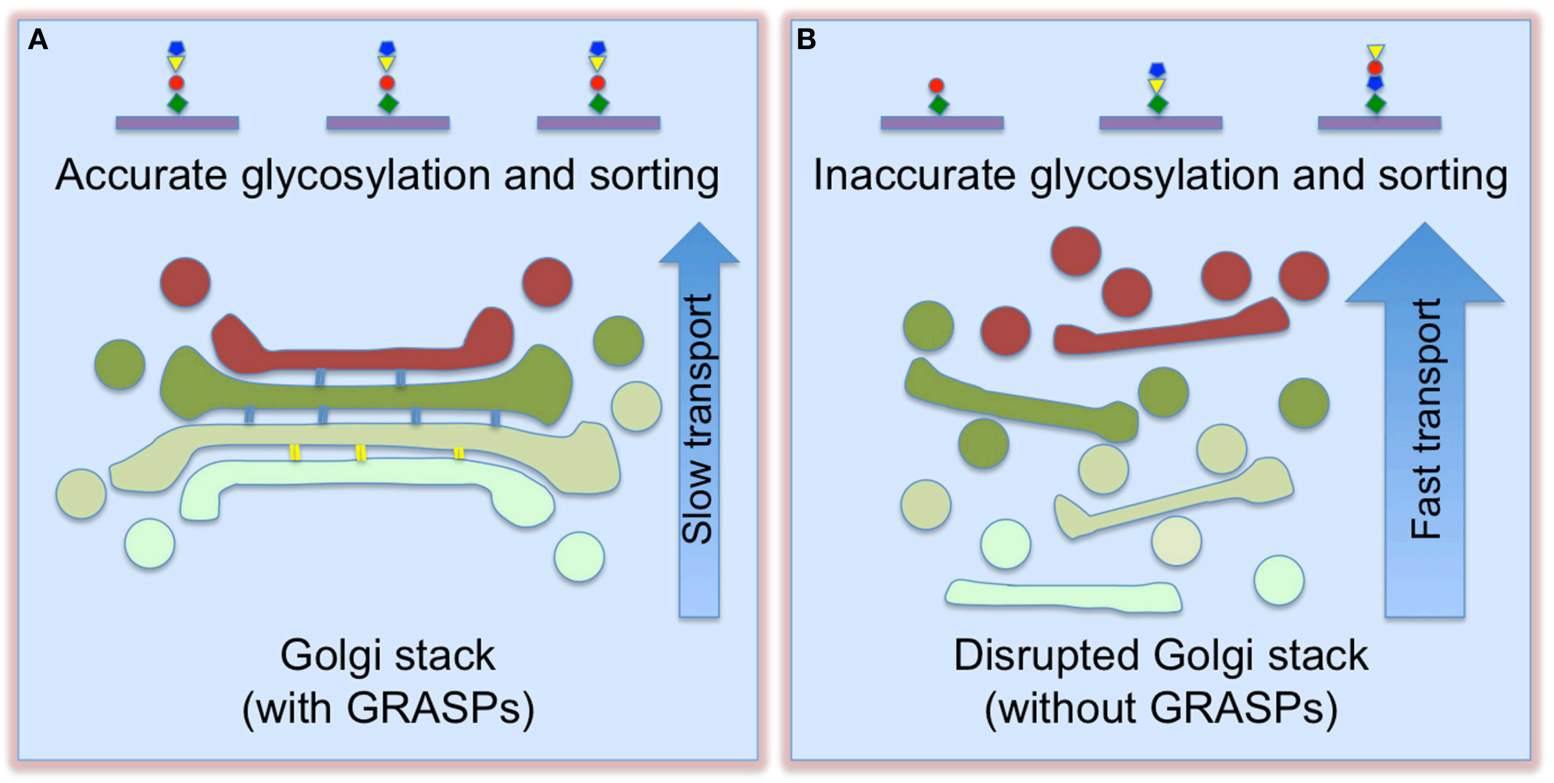
**Golgi destruction accelerates protein trafficking and impairs accurate glycosylation and sorting**. When Golgi cisternae are fully stacked **(A)**, vesicles can only form and fuse at the rims. This slows down trafficking, but enforces accurate glycosylation. Once the cisternae are unstacked **(B)**, more membrane surface area becomes accessible for vesicle budding and fusion, thereby increasing cargo transport. This, however, causes glycosylation and sorting defects (adapted and modified from Xiang et al., 2013).

The best way to answer these questions is to disrupt the Golgi stacks and assess the subsequent effects. One surprising observation is that Golgi destruction accelerates protein trafficking. Inhibition of stacking by microinjecting GRASP65 antibodies resulted in accelerated CD8 trafficking (Wang et al., 2008). Golgi destruction by depletion of both GRASPs enhanced trafficking of the vesicular stomatitis virus G glycoprotein (VSV-G), the cell adhesion protein integrin, and the lysosomal enzyme cathepsin D (Xiang et al., 2013). How could Golgi unstacking enhance protein trafficking? A plausible explanation is that unstacking increases the accessibility of coat proteins to Golgi membranes for vesicle budding and fusion, thereby increasing the rate of protein transport (Figure 2). Indeed, Golgi unstacking increased the rate and efficiency of COPI vesicle formation *in vitro* (Wang et al., 2008), while GRASP-depletion enhanced membrane association of coat proteins in cells (Xiang et al., 2013). In a similar study, the Rothman group reported that GRASP55/65 depletion increased CD8 transport (Lee et al., 2014). In Alzheimer's disease, Golgi fragmentation resulted from GRASP65 phosphorylation by activated Cdk5 enhances APP trafficking and increases Aβ production, which could be reversed by expressing non-phosphorylatable GRASP proteins (Joshi et al., 2014, 2015; Joshi and Wang, 2015).

Golgi destruction impairs accurate protein glycosylation and sorting. GRASP depletion resulted in decreased sialic acid on the cell surface, but the expression level and localization of Golgi enzymes did not significantly change (Xiang et al., 2013). This effect was confirmed by analysis of individual glycoproteins, flow cytometry of cells surface-stained by fluorescent lectins, and glycomic studies. In addition, GRASP depletion also caused missorting of cathepsin D precursor to the extracellular space (Xiang et al., 2013). These results indicate that Golgi structure formation is required for accurate protein glycosylation and sorting. One reasonable explanation is that stacking controls the sequence and speed of protein transport through the Golgi, allowing the cargo to remain in each sub-compartment for a sufficient time period to ensure proper glycosylation in the stack and proper sorting at the TGN; unstacking increases the membrane surface for vesicle formation and protein transport, but causes glycosylation and sorting defects (Figure 2).

An alternative explanation for the glycosylation defects caused by GRASP depletion is Golgi ribbon unlinking. It has been reported that acute inactivation of GRASP65 or GRASP55 led to a loss of *cis*- or *trans*-Golgi integrity, respectively. When one GRASP protein was substituted by the other, the Golgi ribbon was intact, but the membranes were decompartmentalized and glycosylation became defective. Thus, each GRASP plays a cisterna-specific role in linking ministacks to ensure Golgi compartmentalization, enzymes localization, and proper glycosylation (Jarvela and Linstedt, 2014). Additionally, cells from the GRASP65 knockout mouse also showed defects in *cis*-Golgi integrity and glycosylation in the plasma membrane (Veenendaal et al., 2014).

In addition to the role in Golgi structure formation, GRASPs have been implicated in transport of specific cargo, such as TGFα (Kuo et al., 2000), p24 (Barr et al., 2001), CD83 (Stein et al., 2015), CD8α, and Frizzled4 (D'Angelo et al., 2009). These proteins contain a C-terminal hydrophobic tail in which a critical valine residue interacts with the PDZ domain of the GRASP proteins. Here GRASPs function as cargo receptors or chaperones for these transmembrane proteins.

## Conclusions and future directions

Significant progress has been made in the last few years on the GRASP proteins, including their biochemical properties, phosphorylation regulation, crystal structures, and interacting partners, which support GRASPs as the best candidates for Golgi stacking factors. However, there have been discrepancies on their roles in Golgi structure formation mostly resulting from RNAi depletion experiments. A complete knockout of both GRASPs is needed to evaluate their functions. Regardless of the discrepancies, it is generally agreed that (1) GRASPs are important Golgi structural proteins, (2) GRASP proteins may have multiple functions, and (3) there may be other proteins involved in Golgi structure formation. The use of GRASPs as tools to manipulate Golgi stacks has made it possible to assess the biological significance of stack formation. Significantly, there has been an increasing number of human diseases in which Golgi fragmentation has been observed, including autoimmune diseases (Fritzler et al., 1984; Bizzaro et al., 1999), congenital disorders of glycosylation (CDGs) (Durand and Seta, 2000; Freeze and Ng, 2011), cancer (Dennis et al., 1999; Ono and Hakomori, 2004; Tang et al., 2011), as well as Huntington's (Hilditch-Maguire et al., 2000), Parkinson's (Mizuno et al., 2001), and Alzheimer's (Joshi et al., 2014) diseases. Very few studies have attempted to correlate GRASP expression and modifications with Golgi structure and function in different tissues and diseases. Thus, further investigation of the mechanism and significance of Golgi structure formation and the role of GRASPs in Golgi structure assembly may provide meaningful insights into disease therapy.

### Conflict of interest statement

The authors declare that the research was conducted in the absence of any commercial or financial relationships that could be construed as a potential conflict of interest.
